# Age-Related Macular Degeneration: What Do We Know So Far?

**DOI:** 10.15388/Amed.2021.28.1.7

**Published:** 2021-01-18

**Authors:** Ho Hin Ma, Rasa Liutkevičienė

**Affiliations:** Lithuanian University of Health Sciences, Medical Academy, Kaunas, Lithuania; Neuroscience Institute, Lithuanian University of Health Sciences, Medical Academy, Kaunas, Lithuania

**Keywords:** Age-related macular degeneration, drusen, macula lutea, treatment, ageing, genes

## Abstract

Ageing is a natural process that everyone experiences and nobody is an exception. With ageing, our body experiences physiological changes. In this article, the focus is made on the physiological changes of our eyes related to ageing and age-related macular degeneration (AMD), which is the most common cause of incurable visual impairment in developed countries. With ageing populations increasing in many countries, more and more patients will have AMD in a foreseeable future. In Eastern Europe, blindness due to AMD, currently, is approximately 20% and there has been an increasing trend depicted in the future. Generally, AMD can be divided into early stages and two forms in an advanced (late) stage. Advanced AMD form includes neovascular AMD (wet) and geographic atrophy (late dry), both of these are associated with substantial, progressive visual impairment. The pathogenesis of AMD is complex and, by far, not completely understood. Multiple factors have been studied, for example: environmental factor, genetic factor (complement factor H), lifestyle. It has been proved that they are linked to higher the risk of developing of AMD, however, the actual pathogenesis is not yet formulated. AMD progression can also be a culprit to certain biochemical events and molecular changes linked to inflammation and pathological angiogenesis. In nowadays, we do have diagnostic methods for both early and late forms of AMD as well as ways to prevent progression of early AMD and wet AMD. However, until now, there is still no treatment for dry AMD. This article is a brief review of AMD and may hopefully lead to some future directions in early diagnostic methods and treating dry AMD.

## Abstract (lt)

**Amžinė geltonosios dėmės degeneracija: ką žinome iki šiol?:**

**Santrauka.** Senėjimas yra natūralus visų organizmų procesas ir, deja, nėra išimčių. Senstant mūsų kūnas patiria fiziologinių pokyčių.

Šiame straipsnyje daugiausia dėmesio skiriama fiziologiniams akių pokyčiams, susijusiems su senėjimu, tai yra su amžine geltonosios dėmės degeneracija (AGDD), kuri yra labiausiai paplitusi neišgydomo regėjimo sutrikimo priežastis išsivysčiusiose šalyse. Daugelyje šalių senėjanti populiacija didėja, taigi artimoje ateityje AGDD sirgs vis daugiau pacientų. Rytų Europoje aklumas dėl AGDD dabar yra maždaug 20 proc., o ateityje, manoma, didės.

AGDD formos – pradinė ir vėlyvoji. Vėlyvoji AGDD skirstoma į neovaskulinę AGDD (šlapiąją) ir geografinę atrofiją (sausąją), abi yra susijusios su dideliu progresuojančiu regos aštrumo sutrikimu. AGDD patogenezė yra sudėtinga ir iki šiol nėra iki galo aiški. Manoma, kad jos etiologija daugiaveiksnė ir ją lemia: aplinkos veiksniai, genetiniai veiksniai, pavyzdžiui, komplemento faktorius H, gyvenimo būdas, ir įrodyta jų sąsaja su didesne AGDD išsivystymo rizika. AGDD progresavimas galimas ir dėl tam tikrų biocheminių ir molekulinių pokyčių, susijusių su uždegimu ir patologine angiogeneze. Šiais laikais yra ankstyvosios ir vėlyvosios AGDD formų diagnostikos metodų, taip pat būdų kontroliuoti ankstyvosios ir eksudacinės AGDD progresavimą. Tačiau iki šiol vis dar nėra geografinės AGDD gydymo būdų.

Šiame straipsnyje apžvelgiami pagrindiniai AGDD aspektai ir tikimasi, kad tai paskatins mokslininkus kurti ankstyvus atrofinės AGDD diagnostikos būdus ir gydymo kryptis ateityje.

**Raktažodžiai:** amžinė geltonosios dėmės degeneracija, drūzos, geltonoji dėmė, gydymas, senėjimas, genai.

## Introduction

It is commonly known that in course of ageing many eye disorders can develop. In our eyes, there is an innermost layer called retina. Macula is a pigmented area located near the centre of the retina, and is responsible for sharp, clear, straight-ahead vision. Despite the peripheral location, macula is part of the central nervous system. As ageing goes, macula experiences structural and blood flow changes that can predispose patients to age-related macular degeneration (AMD). However, one important thing to notice is that advanced age does not necessarily lead to occurrence of AMD. 

Every cell in the body experiences changes, for example, the accumulation of lipofuscin within lysosomal compartment of postmitotic cells is a sign of ageing due to iron-catalysed oxidative processes in eyes [1], which prevents phagocytosis of obsolete photoreceptors and results in degeneration of photoreceptors in the periphery of retina [2]. Lipofuscin accumulation does not depend on chronological, rather physiological age [3], in other words, factors affecting our physiology – which implies that diet, genetics, metabolism, lifestyle and habits play a role in age pigment levels in our bodies. Induction by reactive oxygen species (ROS) is considered as one of the contributing factors causing AMD [4,5]. N-Retinylidene-N-Retinylethanolamine (A2E) is a major component found in lipofuscin [6], and the mechanisms behind how A2E leads to RPE cells damage and macular degeneration have lately been proposed [7,8]. 

In general, there are two types of AMD, one is called dry AMD, while another one is called wet AMD. Compared with wet AMD, the deteriorating process of dry AMD is slower and visual acuity is better preserved [9]. Nevertheless, 20% of legal blindness over the population of AMD belongs to dry AMD [10], which is considerably significant. Atrophic age related macular degeneration (atrophic AMD), also known as dry AMD, makes up approximately 90% of individuals with conditions of macular degeneration [11]. It is better to include this type of classification: early AMD is defined as the presence of drusen and retinal pigmentary abnormalities; late AMD includes dry AMD (geographic atrophy of the retinal pigmentary epithelium in the absence of neovascular AMD) or neovascular AMD (detachment of the retinal pigment epithelium, hemorrhages, and/or scars). In most cases AMD starts as the dry type and in 10–20% of individuals, it progresses to the wet type [12]. There are various and numerous studies about age-related macular degeneration; however, much of these existing studies and statistics do not indicate whether the results are obtained for ‘wet’ AMD or ‘dry’ AMD specifically. Dry AMD also known as nonneovascular AMD or nonexudative AMD, because there is an absence of abnormal blood vessels growing underneath the retina, leading to leakage of blood or serum. Rather, dry AMD is associated with degeneration of retinal pigment epithelium (RPE) cells and photoreceptor cells, and diagnosed and characterised by the formation and growth of small yellow deposits known as drusen, which dysfunction and damage the Retinal Pigment Epithelium cells, continuation of rod loss and followed by degeneration of cones and eventually only cones of the fovea remain and die off [13]. Moreover, leakage of fluid beneath RPE and Bruch membrane may occur [14].

Upon reaching the phase of losing of choriocapillaris, RPE, and photoreceptors, the stage is known as geographic atrophy, leading to irreversible loss of vision. Geographic Atrophy is an advanced form of dry AMD and characterised by the presence of sharply demarcated atrophic outer retina. To date, although there are no approved medical treatments for both dry AMD and geographic atrophy, some are undergoing clinical trials [15]. Dry AMD can not only develop into geographic atrophy, but 4–12% of cases per year of dry AMD also can develop to choroidal neovascularization (CNV), a characteristic of wet AMD [16], due to the cracking of more fragmented and calcified Bruch’s membrane [17].

In this article, we review the predisposed risk factors of AMD and its etiopathogenesis in order to deduce possibilities of early diagnosis, treatment, and prevention of both early and advanced age-related macular degeneration.

## Composition of drusen

Drusen are composed of lipids, RPE cells fragments, lipofuscin, dendritic cells, potent antigen-presenting cells [18]. In addition, hydroxyapatite (HAP) spherules [19], HAP nodules, trace elements like Zinc and Copper are also found [20]. All of these can be a reason how drusen form and increase in size. Nevertheless, interestingly, they are made up of various proteins, for example: glycoproteins [21], chaperone proteins, Apolipoprotein E, vitronectin, amyloid P, C5, and C5b09 complement complex. Taken together, the information shows that drusen share some features of proteins misfolding in other neurodegenerative conditions [22].

**Figure 1.**
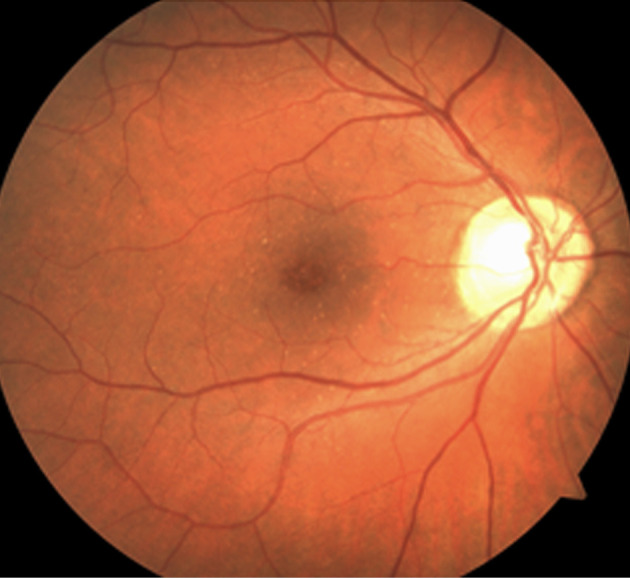
AMD (hard drusen in the fovea) in the right eye

Drusen can be categorized into six types and they are as follows: hard (nodular) drusen, soft (exudative) drusen, basal linear deposits (diffuse drusen or BLinD), cuticular drusen (previous term: basal laminar drusen or BLamD), and reticular pseudodrusen (reticular drusen, subretinal drusenoid deposits). Universally, diffuse drusen and early cuticular drusen are defined as thresholds of the onset of early age-related macular degeneration [23]. Hard drusen (Fig.1) are small, yellow nodules and there is a possibility for them to progress to****dry AMD [24]. They are observed in both normal ageing eyes and AMD patients [25], while soft drusen appear as larger with indistinguishable borders, which can lead to certain parts of RPE being separated [24].

**Figure 2.**
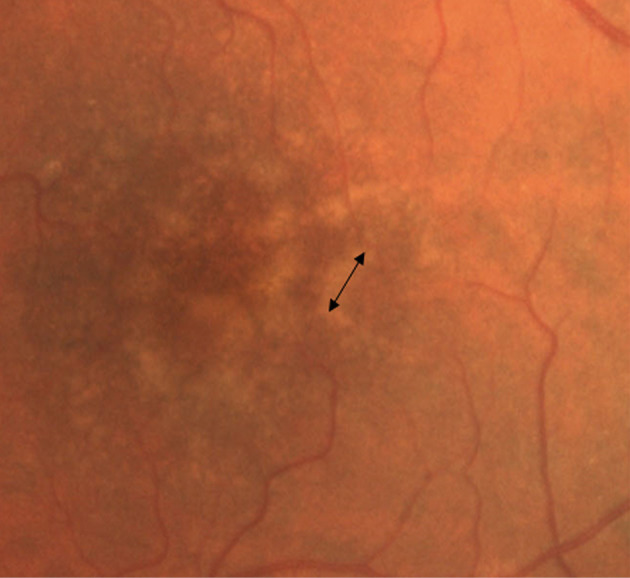
AMD (soft drusen in the fovea with shown diameter of the drusen)

Drusen themselves do not cause any forms of AMD, but their presence in time course definitely increase the risk of developing of AMD. Human drusen consist of lipids primarily derived from photoreceptor cells and serum, and proteins apparently primarily derived from choroidal cells and serum [26]. To date, it is not known how drusen occur, their pathogenesis and ability to vary in location still remain a mystery. Some proposed that formation of drusen can be traced back to the fact that phagocytosis of RPE declines with age [27], and it’s believed that drusen is created by lipofuscin from the oxidative stress accumulated between Bruch’s membrane and (diseased) RPE [28,29].

## Macula lutea

Macula lutea, in homo sapiens, has a diameter approximately 5.5 mm and is subdivided into umbo, foveola, foveal avascular zone, fovea, parafovea, and perifovea regions. It is located at the posterior pole of the eye on the retina and is responsible for central vision. Photoreceptor density is above average throughout macula, therefore when experiencing AMD, central vision is usually faded out or lost. Macula houses three pigments including lutein (L), zeaxanthin (Z) and meso-zeaxanthin (MZ). All of these carotenoids can help in vision and possibly protection from AMD due to their antioxidant properties [30]. Macular carotenoids can be obtained via diet, in other words, the risk of developing of advanced AMD can be reduced by having a diet with macular carotenoids [31], for example, in green leafy vegetables, eggs, corn, red peppers, and some other red, yellow, orange, and dark green fruits and vegetables [31]. Macular carotenoids cannot be synthesized de novo, therefore have to be obtained through diet. Potentially, low levels of serum carotenoids can be a signal alerting someone might be associated with a higher risk of having wet AMD and meanwhile, serum carotenoids can be used as a biomarker of early onset of both dry and wet AMD [32]. Interestingly, there is a positive correlation between levels of carotenoids and neuro-cognitive functions [33], and neuro-cognitive function is less affected when retinal carotenoids are higher even though ageing proceeds. Benefits of retinal carotenoids are not only limited to slow down the decline of neuro-cognitive functions, but also decline the probability of AMD development and advancement [34,35,36] and demonstrate anti-oxidative and anti-inflammatory properties [37,38]. Besides obtaining retinal carotenoids through diet, dietary polyphenols (DPs) have also been reported to be advantageous for our vision [39].

Macula houses cone cells, which are responsible for colour vision under bright light conditions. In dry AMD, cones appear more likely to survive compared to rods****[40], and like in other retinal diseases, rod cells’ apoptosis precedes cone cells’ death. It has been found that abnormalities in distal cone axons could be an indicator to early AMD [41]. AMD is a multifactorial disease of ageing. Many researchers have already proposed the pathogenesis of AMD, and it is summarized as follows: lipofuscin accumulation and retinal epithelial cell damage [42], oxidative stress [43], lipids and lipid peroxidation products [44], chronic inflammation [45], abnormal extracellular matrix [46], and metabolic distress [47]. The risk factors for AMD include: oxidative stress [48], age-related changes in ocular hemodynamics [9], individuals with myocardial infarction [84], genetic predisposition [49], gender [50], ethnicity [51,52,53], colour of iris [54], prolonged exposure of sunlight [55], smoking [56], hypertension [57,58], eating habits [59,60], serum antioxidants and those in diet [61], increased inflammatory markers in blood [62], presence of AMD in the other eye [63], lack of exercise [64], vigorous exercise [65], and accumulation of lipofuscin [66]. Psychological stress is potentially a risk factor for AMD [67]. To date, there is no increased risk of progression to dry AMD after post-cataract surgery [68,69]. Some research showed that blue light damages retina because of higher photon energy [70,71], and it’s true; however, the blue light from electronic devices is dim and therefore, exposure of blue light from electronic devices is not considered as a risk to advance the AMD development. As an old saying goes, “prevention is better than cure”, researchers show that weight loss can prevent the development of AMD [72]; however, it still takes time to see whether there is a reverse relationship, in other words, whether****weight gain will increase the risk of AMD is not yet known and needs to be investigated [73].

Generally, it is agreed that the appearance of drusen deposits at the base of the RPE is the sign of early AMD. Studies [19,20] have found that there is a linkage between calcium and early onset of AMD and the formation of drusen; however, there is inconsistency in their results [74,75]; whether calcium intake can raise or lower the risk, it’s yet to be concluded. To date, studies have been reported that mice are not good animal models to study dry AMD because drusen are rarely seen in mice owing to the simple structure of their basement membrane and lipofuscin production process is different compared with that in humans. For animal models, it’s been reported that *Macaca fascicularis* [76] and *Macaca mulatta* [77] are beneficial in studying the developmental process and formation of drusen in dry AMD. Notably, there is no reported advancement of AMD observed in non homo sapiens primates [78]. Perhaps, by studying the genomics of the animal models mentioned before, certain roles of genes could be uncovered to halt the progression of dry AMD or even its onset. Dry AMD in no doubt affects the quality of life negatively, for example, a person with dry AMD will experience difficulties in reading, driving, and recognizing faces. Currently, there are no treatments for dry AMD and geographic atrophy, an advancement form of dry AMD; however, there are some clinical trials of dry AMD ongoing [79,80,81] and proposed, all of these sound promising and hopeful. Age-related macular degeneration, as mentioned before, is a multifactorial disease and genetic factors play a role in its development and advancement. 36 genes were discovered and identified to be involved in the risk factors and pathogenesis of AMD [82]. Among them, complement factor H (CFH) was the most significant gene found to be related to AMD [83] and is always brought up in other studies in developing lipoprotein treatment for AMD patients [84], and not limited to understanding the pathology of early AMD [85]. Not long ago, studies discovered and proved that there are another 2 gene candidates, PRMT6 and LSS, to study the functional aspects of geographic atrophy [86]. Moreover, recently in the UK, a genetic approach was adopted in the field of atrophic AMD targeting complement factors (NCT03846193) and so far no results are yet available. With the recent advances in bioengineering [87,88] and stem cells technology [27,89,90,91,92], individuals with exudative AMD are reported to have improvement in their visions. The mechanisms of nonexudative AMD are complex; regardless of challenges mentioned in the study, certain ways with the usage of stem cell derived RPE transplant seems to be a promising solution [92].

## Possible diagnosis and treatment for early AMD and atrophic AMD

To date, there have been several treatments of macular degeneration put into practice****(Subretinal Transplantation of Embryonic Stem Cell–Derived Retinal Pigment Epithelium for the Treatment of Macular Degeneration; Retinal pigment epithelium transplantation: concepts, challenges, and future prospects; Phase I/IIa Clinical Trial of Human Embryonic Stem Cell (hESC)-Derived Retinal Pigmented Epithelium (RPE, OpRegen) Transplantation in Advanced Dry Form Age-Related Macular Degeneration (AMD): Interim Results), and more genetic polymorphisms (association of genetic variants at *CETP*, *AGER*, and *CYP4F2* locus with the risk of atrophic age‐related macular degeneration) have been discovered lately to reveal the interactions of the genes to the AMD pathogenesis.

Currently, there are medications/treatment methods for wet AMD, and it’s definitely possible to identify SNP of wet AMD and most ideally, several biomarkers can be deduced and revealed. Since at the advanced stage AMD develops into either dry AMD or wet AMD, by comparing the SNPs in the patients with those in the healthy group, which have been sorted, possibly and hopefully the SNP of dry AMD and the mystery of ageing can be deduced, with the overlap and elimination of some SNPs. In the past, in patients with AMD the connection has been shown with hypertension, dyslipidemia, as well as diabetes mellitus; all of these can be traced to a common factor - blood type, should be evaluated, as well.

## Discussion

Ageing is not a pathology, but a process that every organism is experiencing. Prevention is better than cure, AMD occurs less often in people who exercise on a regular basis, avoid smoking, and eat nutritious foods including green leafy vegetables and fish. There is an importance to further investigate the processes of normal ageing in the human retina and the changing in the eyes of individuals with AMD. It’s also important to study the correlation between trace elements and AMD regardless inconsistencies mentioned before on the role of trace elements in ageing and AMD progression. For example, in AMD patients, both retinal zinc and iron levels decrease in RPE, hence potentially both of these can be an indicator to invite individuals to have a comprehensive eye exam. Further, confirmation if BLam or Blin deposits (basal laminar deposit, BLamD; basal linear deposit,****BLinD) between the cell membrane and the basement membrane of the RPE appear in individuals is also a way, because these deposits precede the emergence of drusen [93]. Several years ago, research studies demonstrated that projected AMD population by 2020 at an increasing trend (94,95,96), and more recently, on the other hand, studies conducted in the USA [97] and Europe discovered that there is a decreasing prevalence of AMD in regions and continents [98]. By combining the data, one can observe inconsistency in the prevalence of AMD. It is highly suggested and recommended to run a meta-analysis to follow up and unmask and reflect the real phenomenon of AMD in regions and continents nowadays, also to see whether nowadays the prevalence of AMD is declined or actually increased as expected, and whether there is a trend of individuals of developing AMD at a later age or an earlier age. It’s worthy of note that the “A” in AMD refers to the biological age instead of the chronological age. In other words, a healthy lifestyle, a healthy dietary habits, good mental health, stress-free environments [99, 100], not just genetic factors, play a role in slowing down ageing and ageing associated diseases. It is recommended to conduct an investigation whether there is a discrepancy between the biological age and the chronological age in individuals with different stages of AMD, and whether protecting telomeres from damage can treat AMD [8]. An interesting thing is that for 10–20% of patients with dry AMD it develops into wet AMD – how does it develop? What genes play certain roles to make it happen? Can the conversion of dry AMD to wet AMD be a cure for dry AMD? Investigations are recommended to discover the possibilities, forward the discoveries, and unfold the mystery.

## Conclusion

Regardless of various risk factors and candidate genes have been discovered and mentioned above, as well as associations drawn to atrophic AMD, it still makes up the majority cases of age-related macular degeneration. Yet, a broader understanding of how interactions of factors, the causative factors of dry AMD, and correlation between genetics and pathological condition remains a mystery. Ageing is a complicated process with multiple factors, let alone age-related macular degeneration. By far, the pathogenesis, causation and correlation of both environmental and genetic factors of atrophic AMD are awaiting to be discovered and reviewed. Hopefully in the future, a feasible cure and treatment can be provided.
